# Prognostic Utility of the Modified Systemic Inflammation Score for Patients Undergoing Oral Cavity Cancer Surgery

**DOI:** 10.3390/diagnostics14242856

**Published:** 2024-12-19

**Authors:** Ku-Hao Fang, Sheng-Wei Lo, Adarsh Kudva, Andrea De Vito, Yuan-Hsiung Tsai, Cheng-Ming Hsu, Geng-He Chang, Ethan I. Huang, Ming-Shao Tsai, Chia-Hsuan Lai, Ming-Hsien Tsai, Chun-Ta Liao, Chung-Jan Kang, Yao-Te Tsai

**Affiliations:** 1 Department of Otorhinolaryngology-Head and Neck Surgery, Chang Gung Memorial Hospital, Taoyuan 333423, Taiwan; kuhaofang7872@gmail.com (K.-H.F.); liaoct@cgmh.org.tw (C.-T.L.); handneck@gmail.com (C.-J.K.); 2 College of Medicine, Chang Gung University, Taoyuan 330036, Taiwan; russell.tsai@gmail.com (Y.-H.T.); scm0031@cgmh.org.tw (C.-M.H.); genghechang@gmail.com (G.-H.C.); ehuang.mdphd@gmail.com (E.I.H.); b87401061@gmail.com (M.-S.T.); chiahsuan7092@gmail.com (C.-H.L.); b9302094@cgmh.org.tw (M.-H.T.); 3 Department of Otorhinolaryngology-Head and Neck Surgery, Chang Gung Memorial Hospital, Chiayi 613016, Taiwan; dav85263@gmail.com; 4 Department of Oral and Maxillofacial Surgery, Manipal College of Dental Sciences, Manipal Academy of Higher Education, Manipal 576104, India; adarsh.kudva@manipal.edu; 5 Ear Nose Throat (ENT) Unit, Department of Surgery, Forlì Hospital Health Local Agency of Romagna, 47121 Forlì, Italy; drandreadevito@gmail.com; 6 Department of Diagnostic Radiology, Chang Gung Memorial Hospital, Chiayi 613016, Taiwan; 7 Department of Radiation Oncology, Chang Gung Memorial Hospital, Chiayi 613016, Taiwan; 8 Department of Otorhinolaryngology-Head and Neck Surgery, Chang Gung Memorial Hospital, Kaohsiung 833253, Taiwan

**Keywords:** oral cavity cancer, squamous cell carcinoma, modified systemic inflammation score, nomogram, survival outcomes

## Abstract

Background/Objectives: Chronic inflammation significantly contributes to human malignancies. We investigated the prognostic significance of the preoperative modified systemic inflammation score (mSIS) in patients with primary oral cavity squamous cell carcinoma (OCSCC). Methods: We retrospectively reviewed data from 320 OCSCC patients who underwent curative surgery between 2007 and 2017. Based on preoperative lymphocyte-to-monocyte ratio (LMR) and serum albumin levels, patients were classified into three groups: mSIS = 2 (LMR < 3.4 and albumin < 4.0 g/dL), mSIS = 1 (LMR < 3.4 or albumin < 4.0 g/dL), and mSIS = 0 (LMR ≥ 3.4 and albumin ≥ 4.0 g/dL). We explored the associations between the preoperative mSIS and overall survival (OS) and disease-free survival (DFS). We developed a nomogram based on mSIS for OS prediction. Results: The distribution was mSIS = 0 (*n* = 197, 61.6%), mSIS = 1 (*n* = 99, 30.9%), and mSIS = 2 (*n* = 24, 7.5%). Kaplan–Meier estimated OS and DFS for the mSIS = 0, mSIS = 1, and mSIS = 2 groups demonstrated a sequential decrease (both *p* < 0.001). The prognostic significance of mSIS was consistent across subgroup analyses. Multivariable analysis revealed that mSIS = 1 and mSIS = 2 were independent negative prognostic indicators. The mSIS-based nomogram effectively predicted OS (concordance index: 0.755). Conclusions: The mSIS reliably predicts OS and DFS in OCSCC patients undergoing surgery, with the nomogram providing individualized OS estimates, enhancing mSIS’s clinical utility.

## 1. Introduction

Head and neck cancer (HNC) greatly affects the quality of life, imposes a substantial socioeconomic burden, and is the seventh most common malignancy worldwide [1]. Among HNC subsites, oral cavity cancer is the most prevalent [1], with oral cavity squamous cell carcinoma (OCSCC) accounting for over 90% of cases [2]. Various patient-related factors, such as old age, human papillomavirus infection, comorbidities, and the use of tobacco or alcohol, influence the prognoses of OCSCC [3]. Additionally, tumor-specific factors like cancer stage, tumor grade, perineural invasion (PNI) status, extranodal extension (ENE) status, and lymphovascular invasion (LVI) status also play pivotal roles in OCSCC outcomes [4,5]. These tumor-specific factors are typically assessed post-surgery, underscoring the importance of preoperative prognostic markers for early patient stratification and better treatment planning.

Systemic inflammation is critical in cancer development, progression, and metastasis, affecting angiogenesis and extracellular matrix remodeling [6,7]. Cancer-related inflammatory responses have been correlated with declines in nutritional and functional status, impacting survival outcomes [8,9,10]. Several inflammatory biomarkers, such as the lymphocyte-to-monocyte ratio (LMR) and neutrophil-to-lymphocyte ratio (NLR), have been identified as prognostic indicators in OCSCC [11]. Malnutrition, often indicated by low serum albumin levels, is common among OCSCC patients [12] and is linked to poorer overall survival (OS) [13]. Eltohami et al. introduced the systemic inflammation score (SIS) based on median serum albumin and LMR values, linking a higher SIS to lower OS in patients with OCSCC [14]. Lin et al. later proposed a modified systemic inflammation score (mSIS) using specific LMR (3.4) and albumin (4.0 g/dL) thresholds, demonstrating its superior prognostic utility compared to the SIS in predicting OS for gastric cancer patients [15]. Subsequent studies across various malignancies, including lung cancer, thyroid cancer, and adenocarcinoma of the upper digestive tract, have consistently shown the mSIS’s superior utility over SIS in predicting survival outcomes [15,16,17,18]. However, no research has yet examined the preoperative mSIS’s prognostic value in OCSCC. Thus, we conducted a retrospective study to determine whether the mSIS effectively indicates prognosis in OCSCC patients undergoing surgery. Given that the mSIS incorporates both nutritional status and systemic inflammatory response, we anticipated a robust association between the mSIS and prognosis in patients with OCSCC.

## 2. Materials and Methods

### 2.1. Study Cohort

This observational study retrospectively analyzed medical records from 349 consecutive patients who received ablative surgery for OCSCC at our hospital between January 2007 and December 2017. We included individuals with a histopathologically verified diagnosis of OCSCC who had curative surgery as their primary treatment. Patients were excluded if they had unresectable cancer (*n* = 5), synchronous cancer or distant metastasis at diagnosis (*n* = 5), history of cancer (*n* = 8), acute infection in any form prior to surgery, autoimmune disease, hematological disease, or severe hepatic or renal disease (*n* = 4). Additionally, patients who received neoadjuvant therapy (*n* = 5) or lacked laboratory or follow-up data (*n* = 2) were excluded. The final analysis included 320 patients. The study, adhering to the Declaration of Helsinki, was approved by Chang Gung Memorial Hospital’s IRB (IRB number: 202400053B0). The Institutional Review Board waived the requirement for informed consent due to the retrospective nature of the study.

### 2.2. Data Collection

Medical personnel conducted a comprehensive review of the laboratory, demographic, and histopathological data from electronic medical records. Detailed patient information, including age, sex, tumor subsites, pathological characteristics, comorbidities, health-related habits, and treatment modalities, is presented in Table 1. The pathological cancer stage was documented based on the American Joint Committee on Cancer (AJCC) Cancer Staging Manual, 8th Edition (2018). We defined and categorized comorbidities using the Charlson Comorbidity Index (CCI) [19]. In terms of health-related habits, patients were categorized as betel nut consumers if they consumed betel nut daily for at least 1 year. Patients who smoked ≥ 1 packet of cigarettes daily for a minimum of 1 year were categorized as cigarette users, and patients who consumed ≥ 2 alcoholic beverages per week for at least 6 months were categorized as alcohol consumers [4]. Patients were further categorized by the number of habit exposures: none, one, or two or more, corresponding to the absence, presence of one, or presence of two or more of the aforementioned health habits, respectively.

### 2.3. Treatment Protocol

Each patient underwent standard presurgical assessments, including magnetic resonance imaging (MRI) or computed tomography (CT) scans of the head and neck, nuclear bone scans, chest radiographs, panendoscopy, and abdominal ultrasound. All patients then had ablative surgery for primary OCSCC, with neck dissection as needed. Plastic surgeons performed reconstructive procedures to address surgical defects. Indications for adjuvant therapy were outlined in a related study by our colleagues [20]. Adjuvant treatments, when necessary, began within six weeks post-surgery. Cisplatin-based chemotherapy was given weekly at 40 mg/m^2^ or every three weeks at 100 mg/m^2^, depending on the oncologist’s assessment and the patient’s health and preferences. Intensity-modulated radiation therapy was administered at a total dose of 66 Gy, in daily fractions of 2 Gy, five days a week.

### 2.4. Laboratory Measurements

Blood tests were conducted within a week before surgery. Hematological tests, analyzed with a Sysmex SE-9000 hematology analyzer (Kobe, Japan), provided data to calculate the LMR using the formula: LMR = absolute lymphocyte count (per mm^3^)/absolute monocyte count (per mm^3^). Serum albumin levels were measured with a Cobas 8000 biochemistry analyzer from Roche Diagnostics (Rotkreuz, Switzerland). The mSIS was determined using validated cutoff values for LMR (3.4) and albumin (4.0 g/dL): an LMR ≥ 3.4 and albumin ≥ 4.0 g/dL yielded an mSIS of 0; either an LMR < 3.4 or albumin < 4.0 g/dL corresponded to an mSIS of 1, and both LMR < 3.4 and albumin < 4.0 g/dL resulted in an mSIS of 2 [15].

### 2.5. Follow-Up and Study Endpoints

Patients were assessed every two months for the first two years after surgery and every 4–6 months from years two to five. Follow-up included physical examinations, endoscopic evaluations, and laboratory tests. Advanced imaging (CT or MRI) was performed semiannually for the first two years and annually afterward. The follow-up period concluded in December 2020. The primary endpoint was OS, measured from surgery to any-cause mortality or censoring at the last follow-up. The secondary endpoint was disease-free survival (DFS), defined as the time from surgery to locoregional recurrence, distant metastasis, mortality, or censoring at the last follow-up.

### 2.6. Statistical Analysis

We evaluated data distribution normality using the Shapiro-Wilk test. Non-normally distributed continuous variables are shown as median with interquartile ranges, while normally distributed variables are reported as mean with standard deviations. Categorical variables are displayed as counts and percentages. Intergroup differences in clinicopathological features were analyzed using the Mann–Whitney U test for continuous variables and the chi-square test for categorical variables. We constructed survival curves through the Kaplan–Meier method and assessed intergroup survival differences with the log-rank test. We estimated the prognostic values of clinicopathological variables on survival through Cox proportional hazards models, with results presented as hazard ratios (HRs) and 95% confidence intervals (CIs). We included any variable with a *p*-value less than 0.1 in the univariable analysis into the multivariable analysis, and we considered a final two-sided *p*-value of less than 0.05 to indicate statistical significance. We performed all statistical analyses using SPSS, version 21.0 (IBM SPSS, Chicago, IL, USA).

To improve OS prediction using mSIS, we constructed a nomogram with the rms package in R version 5.1-0 (Vanderbilt University, Nashville, TN, USA) [21]. Independent variables identified in multivariable analysis for OS were incorporated in the nomogram, and its predictive accuracy was assessed using the concordance index (C-index) and calibration plots.

## 3. Results

### 3.1. Patient Characteristics

Table 1 summarizes the clinicopathological characteristics of the 320 patients. Most were male (90.3%), with 63.4% younger than 65 years. The median age was 61, with a median follow-up of 41.0 months. Common tumor subsites included the tongue (*n* = 125, 39.1%) and buccal mucosa (*n* = 102, 31.9%). Regarding cancer stage, 110 (34.4%) and 210 (65.6%) patients were determined to have stage I–II and III–IV disease, respectively. Treatment included surgery alone (*n* = 152, 47.5%), surgery with adjuvant radiotherapy (*n* = 44, 13.8%), and surgery with adjuvant chemoradiotherapy (*n* = 124, 38.8%). mSIS scores were 0, 1, and 2 in 197 (61.6%), 99 (30.9%), and 24 (7.5%) patients, respectively.

### 3.2. Association Between Clinicopathological Characteristics and mSIS

Table 1 also details the associations between clinicopathological characteristics and the preoperative mSIS. Higher mSIS was significantly associated with advanced cancer stages, higher T and N status, ENE, need for adjuvant therapy, and depth of invasion (DOI) ≥ 10 mm. No significant associations were found between mSIS and factors such as tumor subsite, sex, age, personal habits, LVI, PNI, closest resection margin, tumor grade, or CCI.

### 3.3. Association Between mSIS and OS

**Figure 1:**
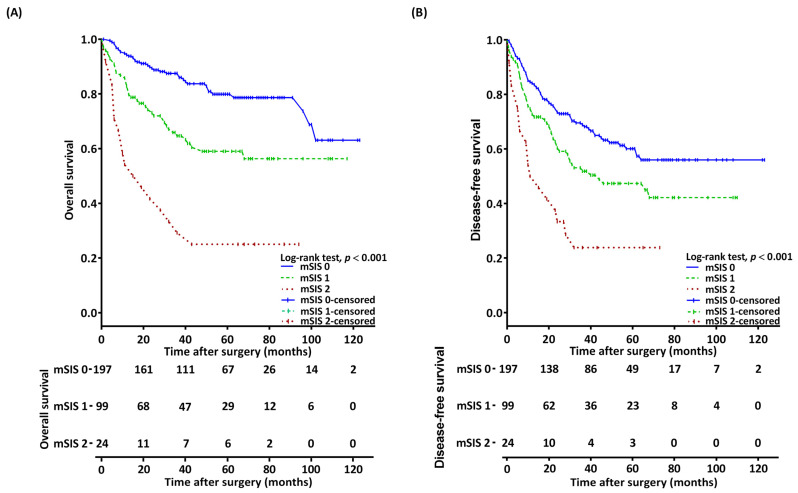
Survival analysis (**A**) Kaplan–Meier overall survival curves for each mSIS group (*p* < 0.001), (**B**) Kaplan–Meier disease-free survival curves for each mSIS group (*p* < 0.001). Abbreviation: mSIS, modified systemic inflammation score.

**Figure 2:**
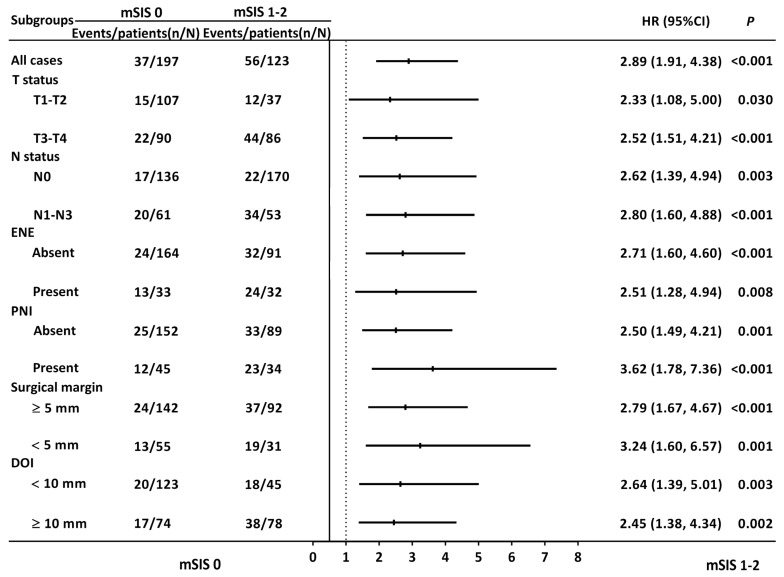
Subgroup analysis of the discriminative ability of mSIS for overall survival. Abbreviations: CI, confidence interval; DOI, depth of invasion; ENE, extranodal extension; HR, hazard ratio; mSIS, modified systemic inflammation score; PNI, perineural invasion.

**Figure 3:**
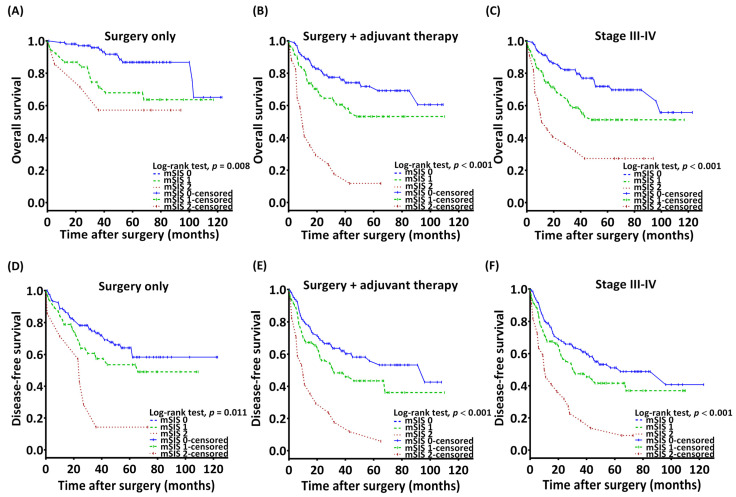
Overall survival curves based on preoperative mSIS in (**A**) patients who received surgery alone (*p* = 0.008), (**B**) patients who received surgery and adjuvant therapy (*p* < 0.001), and (**C**) patients with stage III–IV disease (*p* < 0.001). Disease-free survival curves based on preoperative mSIS in (**D**) patients who received surgery alone (*p* = 0.011), (**E**) patients who received surgery and adjuvant therapy (*p* < 0.001), and (**F**) patients with stage III–IV disease (*p* < 0.001). Abbreviations: mSIS, modified systemic inflammation score.

Kaplan–Meier analysis revealed distinct 5-year OS rates among patients with mSIS scores of 0, 1, and 2, which were 79.9%, 59.1%, and 25.2%, respectively (*p* < 0.001, Figure 1A). OS associations with mSIS remained significant across patient groups defined by T and N status, ENE, PNI, DOI (≥10 mm and <10 mm), and the closest resection margins (≥5 mm and <5 mm; Figure 2). Significant OS differences were observed in both the surgery-alone group (*p* = 0.008, Figure 3A) and the surgery with adjuvant therapy group (*p* < 0.001, Figure 3B). Notable 5-year OS rate differences based on mSIS were also evident among patients with advanced cancer (*p* < 0.001, Figure 3C). Analysis for patients with early-stage disease was omitted due to the small size of this subgroup. Univariable analysis revealed that poorly differentiated (P–D) tumors, stage III–IV disease, PNI, LVI, and mSIS of 1 or 2 were significantly associated with poor OS (Table 2, all *p* < 0.001). Multivariable analysis confirmed mSIS of 1 (HR = 2.021; CI, 1.275–3.204; *p* = 0.003) and mSIS of 2 (HR = 5.095; CI: 2.804–9.258; *p* < 0.001) as independent negative prognostic indicators for OS, alongside stage III–IV disease, LVI, and P–D tumors (Table 2).

### 3.4. Association Between mSIS and DFS

Estimated 5-year DFS rates among patients with mSISs of 0, 1, and 2 were 60.1%, 47.3%, and 12.5%, respectively (*p* < 0.001, Figure 1B). Significant differences in 5-year DFS rates based on mSIS were observed in both the surgery-alone group (*p* = 0.011, Figure 3D) and the surgery with adjuvant therapy group (*p* < 0.001, Figure 3E). Among patients with advanced cancer, notable differences in 5-year DFS rates based on mSIS was also evident (*p* < 0.001, Figure 3F). The univariable analysis demonstrated significant associations between poor DFS and stage III–IV disease, LVI, P–D, and mSIS of 1 or 2 (Table 3). Multivariable analysis confirmed mSIS score of 2 (HR = 3.590; CI, 2.167–5.947; *p* < 0.001), P–D tumors, and stage III–IV disease as independent predictors of DFS.

### 3.5. Predictive Nomogram

**Figure 4:**
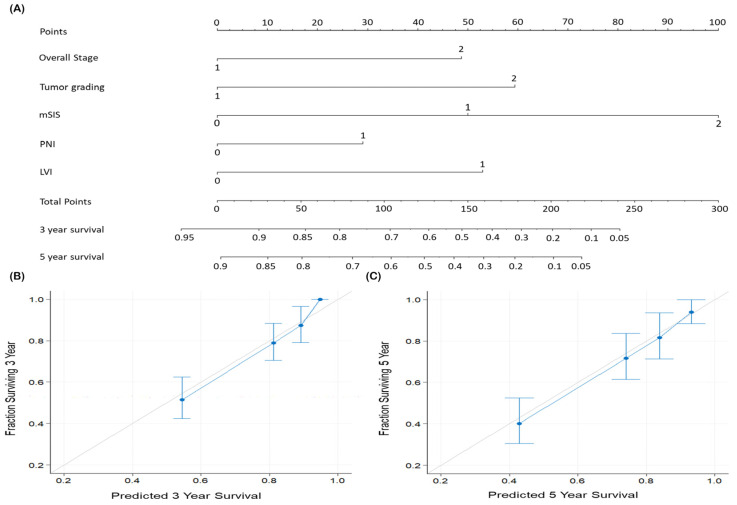
Nomogram for predicting overall survival. (**A**) Independent prognostic factors identified in multivariable analysis are incorporated into the nomogram. Each parameter’s risk contribution is represented by a line segment with corresponding uppermost points. Total points are calculated by summing the points for each parameter. To estimate the likelihood of 3-year and 5-year OS, draw a vertical line downward from the total points. (**B**) Calibration plot for 3-year OS. (**C**) Calibration plot for 5-year OS. An ideal OS prediction is represented by a 45° line, while the nomogram’s predictions are illustrated by a blue line. Predictive performance and 95% confidence intervals are represented by blue dots and bars, respectively. Abbreviations: LVI, lymphovascular invasion; mSIS, modified systemic inflammation score; PNI, perineural invasion.

To quantitatively predict 3-year and 5-year OS rates, we devised a nomogram incorporating cancer stage, tumor grade, mSIS, PNI, and LVI (Figure 4A). For comparison, a nomogram based solely on the AJCC staging system was also constructed. The area under the curve for the developed nomogram was 0.748 (sensitivity, 68.6%; specificity, 77.4%). The mSIS-based nomogram achieved a C-index of 0.755 (95% CI, 0.723–0.786), outperforming an AJCC stage-based nomogram (C-index = 0.628; 95% CI, 0.602–0.655). Calibration plots for assessing the congruence between actual 3-year and 5-year OS rates and nomogram-predicted OS showed high accuracy (Figure 4B,C), indicating that the mSIS-based nomogram reliably predicts OS in patients with OCSCC.

## 4. Discussion

This is the first study to explore the prognostic utility of mSIS in surgically treated OCSCC patients. Our analysis revealed that high preoperative mSIS scores correlate with unfavorable clinicopathological features, such as advanced cancer stage, high T and N status, ENE, and DOI ≥ 10 mm. Additionally, high mSIS scores were associated with adjuvant therapy needs and shorter survival. These results suggest that preoperative systemic inflammation and poor nutritional status, reflected by mSIS, are associated with OCSCC aggressiveness. Kaplan–Meier analysis showed that a high mSIS is associated with lower 5-year OS and DFS rates, with subgroup analyses confirming a significant and consistent link between mSIS and OS across various pathological subgroups. Multivariable analysis confirmed mSIS = 2 as an independent risk factor for both DFS and OS, while mSIS = 1 was an independent risk factor for OS alone. Overall, mSIS is a cost-effective prognostic indicator for OCSCC, aiding in the identification of patients who may benefit from comprehensive therapeutic approaches, including nutritional support, side effect management, and rigorous follow-up [22].

In clinical practice, the AJCC staging system is widely used for patient prognostication and treatment planning. The most recent edition has evolved to incorporate ENE and DOI [23]. However, it still excludes several significant clinicopathological factors, such as PNI, LVI, and host immune-nutrition status [24,25]. Nomograms, which incorporate various prognostic factors, are frequently employed for survival prediction in cancer patients [4]. We developed a nomogram that includes mSIS, cancer stage, tumor grade, and PN/LVI status to enhance OS prediction. After surgery, this nomogram can be applied to patients with OCSCC based on their pathological reports, providing reliable OS predictions and assisting in adjuvant therapy decisions.

Evidence increasingly indicates that inflammation-based biomarkers, such as NLR and LMR, are linked to prognosis in patients with OCSCC [26]. However, these markers can be influenced by host conditions, and arbitrary determination of cutoff values can impact their general applicability. The SIS, which incorporates serum albumin and LMR to reflect systemic inflammation and nutritional status, was first introduced as a prognostic tool in colorectal cancer and clear cell renal carcinoma [27,28]. Nonetheless, the SIS’s generalizability is limited due to varying cutoff values for LMR and albumin across studies. In contrast, the mSIS employs consistent cutoff values, enhancing its reliability as an immune- and nutrition-based prognostic tool for various cancers. For instance, in a study of 317 patients with adenocarcinoma of the esophagogastric junction, mSIS was found to be an independent predictor of OS and relapse-free survival [29]. Similarly, a study of 443 patients with esophageal cancer showed mSIS to be an effective predictor of DFS and hematogenous recurrence [30]. When compared to its components (LMR and albumin) and the original SIS, mSIS proved to be a more accurate predictor of OS in patients with gastric cancer [15,29]. Furthermore, a study on indeterminate thyroid biopsies found that a higher mSIS correlated with increased thyroid malignancy risk, suggesting a potential diagnostic role for the mSIS [16]. These findings highlight mSIS’s effectiveness in cancer management. Future research could explore its clinical utility, particularly in patients undergoing targeted or immunotherapy, to broaden understanding of its potential benefits.

The exact mechanisms behind the prognostic value of mSIS in OCSCC patients are not yet fully understood. Lymphocytes—such as CD4+ type 1 T helper lymphocytes, CD8+ cytotoxic T lymphocytes, and natural killer T-cells—serve as the frontline of antitumor defense [31]. They detect cancer cells, induce apoptosis, and inhibit both angiogenesis and metastasis [32,33]. As cancer progresses, the numbers of natural killer T-cells and tumor-infiltrating CD8+ T-cells decrease, indicating a breakdown in immunosurveillance [34]. This reduction in lymphocyte count may signal issues within the immune system. In contrast, monocytes contribute to tumor progression by inhibiting CD8+ T-cell activity and promoting regulatory T-cell recruitment [35]. The monocytes can also transform into tumor-associated macrophages, aiding in extracellular matrix remodeling, angiogenesis, and the intravasation of cancer cells, thus facilitating tumor spread [36,37]. Therefore, a low LMR due to high monocyte or low lymphocyte counts may indicate a weakened host immune system and a poorer prognosis [38]. Albumin, the most abundant protein in human serum, helps transport various substances, neutralize free radicals, and stabilize DNA replication [39]. In cancer patients, tumor-related cytokines like tumor necrosis factor-α and interleukin-6 can hinder albumin gene transcription in liver cells, leading to hypoalbuminemia [40,41]. Therefore, hypoalbuminemia could be indicative of reduced antitumor immunity [42]. Malnutrition and hypoalbuminemia are common in patients with OCSCC [43], and both are associated with poorer prognoses [44]. However, the prognostic significance of LMR and albumin is inconsistent across studies in the literature. Lee et al. conducted a study involving 291 patients with OCSCC, using ROC curve analysis to identify the optimal cutoffs for LMR and albumin [45]. Their findings indicated that LMR and albumin were not independent prognostic factors within their study context. Instead, the mSIS, which incorporates validated cutoffs for both LMR and albumin, offers a potentially viable alternative for integrating these parameters into OCSCC management rather than relying on either one individually. While these findings shed light on the link between mSIS and survival in patients with OCSCC, the exact underlying mechanisms require further investigation.

The study’s strengths include a large, uniformly treated OCSCC cohort and extended follow-up. Unlike other biomarkers with inconsistent cutoff values [46], mSIS is calculated using stable serum albumin and LMR cutoffs, enhancing its reproducibility in clinical settings. However, the study has limitations, including its retrospective, single-center design, which may introduce selection bias. The prognostic value of mSIS was not validated with an independent dataset, and the potential effects of anti-inflammatory agents or nutritional supplements were not considered. Previous research has shown that mSIS measured pre-surgery is a better prognostic indicator than mSIS measured before neoadjuvant treatment in esophageal cancer [30]. Our study excluded patients who received neoadjuvant therapy, so we could not assess mSIS’s prognostic utility in this subgroup. Further large-scale, multicenter prospective trials are needed to confirm our findings.

## 5. Conclusions

Our findings underscore the value of the preoperative mSIS as a straightforward yet powerful prognostic tool for patients undergoing surgery for OCSCC. Incorporating mSIS with significant clinicopathologic factors in the nomogram improves OS prediction accuracy and supports its clinical application. Further prospective multicenter studies are necessary to validate our findings before the mSIS and the nomogram can be widely adopted in clinical practice.

## Figures and Tables

**Table 1:** Baseline characteristics of 320 patients stratified by preoperative mSIS.

Variable	Total, *n* (%)	mSIS 0 (*n* = 197)	mSIS 1 (*n* = 99)	mSIS 2 (*n* =24)	*p* Value
Age (years)					0.500 ^a^
<65	203 (63.4%)	129 (65.5%)	61 (61.6%)	13 (54.2%)	
≥65	117 (36.6%)	68 (34.5%)	38 (38.4%)	11 (45.8%)	
Sex					0.197 ^a^
Men	289 (90.3%)	178 (90.4%)	87 (87.9%)	24 (100.0%)	
Women	31 (9.7%)	19 (9.6%)	12 (12.1%)	0 (0.0%)	
Tumor location					0.079 ^a^
Tongue	125 (39.1%)	85 (43.1%)	31 (31.3%)	9 (37.5%)	
Buccal mucosa	102 (31.9%)	61 (31.0%)	30 (30.3%)	11 (45.8%)	
Gingiva	42 (13.1%)	21 (10.7%)	20 (20.2%)	1 (4.2%)	
Retromolar trigone	19 (5.9%)	12 (6.1%)	5 (5.1%)	2 (8.3%)	
Lip	13 (4.1%)	6 (3.0%)	7 (7.1%)	0 (0.0%)	
Mouth floor	13 (4.1%)	6 (3.0%)	6 (6.1%)	1 (4.2%)	
Hard palate	6 (1.9%)	6 (3.0%)	0 (0.0%)	0 (0.0%)	
Personal Habits *					0.130 ^a^
No exposure	38 (11.9%)	27 (13.7%)	11 (11.1%)	0 (0.0%)	
One exposure	18 (5.6%)	8 (4.1%)	9 (9.1%)	1 (4.2%)	
Two or all exposure	264 (82.5%)	162 (82.2%)	79 (79.8%)	23 (95.8%)	
AJCC stage					<0.001 ^a^
I–II	110 (34.4%)	83 (42.1%)	25 (25.3%)	2 (8.3%)	
III–IV	210 (65.6%)	114 (57.9%)	74 (74.7%)	22 (91.7%)	
T status					<0.001 ^a^
T1–T2	144 (45.0%)	107 (54.3%)	32 (32.3%)	5 (20.8%)	
T3–T4	176 (55.0%)	90 (45.7%)	67 (67.7%)	19 (79.2%)	
N status					0.019 ^a^
N0	206 (64.4%)	136 (69.0%)	60 (60.6%)	10 (41.7%)	
N+	114 (35.6%)	61 (31.0%)	39 (39.4%)	14 (58.3%)	
Presence of PNI					0.614 ^a^
No	241 (75.3%)	152 (77.2%)	72 (72.7%)	17 (70.8%)	
Yes	79 (24.7%)	45 (22.8%)	27 (27.3%)	7 (29.2%)	
Presence of ENE					0.040 ^a^
No	255 (79.7%)	164 (83.2%)	76 (76.8%)	15 (62.5%)	
Yes	65 (20.3%)	33 (16.8%)	23 (23.2%)	9 (37.5%)	
Presence of LVI					0.050 ^a^
No	298 (93.1%)	188 (95.4%)	90 (90.9%)	20 (83.3%)	
Yes	22 (6.9%)	9 (4.6%)	9 (9.1%)	4 (16.7%)	
Tumor grading					0.540 ^a^
W–D/M–D	283 (88.4%)	177 (89.8%)	86 (86.9%)	20 (83.3%)	
P–D	37 (11.6%)	20 (10.2%)	13 (13.1%)	4 (16.7%)	
Closest resection margin					0.867 ^a^
≥5 mm	234 (73.1%)	55 (27.9%)	25 (25.3%)	6 (25.0%)	
<5 mm	86 (26.9%)	142 (72.1%)	74 (74.7%)	18 (75.0%)	
DOI ≥ 10 mm					<0.001 ^a^
No	168 (52.5%)	123 (62.4%)	39 (39.4%)	6 (25.0%)	
Yes	152 (47.5%)	74 (37.6%)	60 (60.6%)	18 (75.0%)	
Treatment modality					0.001 ^a^
Surgery only	152 (47.5%)	107 (54.3%)	38 (38.4%)	7 (29.2%)	
Surgery then RT	44 (13.8%)	27 (13.7%)	17 (17.2%)	0 (0.0%)	
Surgery then CRT	124 (38.8%)	63 (32.0%)	44 (44.4%)	17 (70.8%)	
CCI					0.255 ^a^
0	171 (53.4%)	109 (55.3%)	53 (53.5%)	9 (37.5%)	
≥1	149 (46.6%)	88 (44.7%)	46 (46.5%)	15 (62.5%)	
WBC (×10^3^/μL), median (IQR)	7.8 (6.3–9.7)	7.5 (6.2–9.2)	8.8 (6.4–10.6)	9.9 (7.8–11.9)	0.093 ^b^
Lymphocyte (×10 ^3^/μL), median (IQR)	2.03 (1.59–2.59)	2.30 (1.85–2.83)	1.78 (1.37–2.09)	1.57 (1.10–2.03)	<0.001 ^b^
Monocyte (×10 ^3^/μL), median (IQR)	435.1 (340.8–580.7)	388.8 (322.2–490.9)	560.9 (386.9-678.6)	595.7 (447.3–752.1)	<0.001 ^b^
Albumin (g/L), median (IQR)	44.3 (41.8–46.7)	45.70 (43.7–47.2)	42.01 (38.0–45.0)	35.85 (31.3–38.2)	<0.001 ^b^
LMR, median (IQR)	4.50 (3.3–6.1)	5.40 (4.5–6.8)	3.10 (2.6–3.9)	2.70 (2.1–3.1)	<0.001 ^b^

Abbreviations: AJCC, American Joint Committee on Cancer; CCI, Charlson Comorbidity Index; CRT, chemoradiotherapy; DOI, depth of invasion; ENE, extranodal extension; IQR, interquartile range; LVI, lymphovascular invasion; M–D, moderately differentiated; P–D, poorly differentiated; PNI, perineural invasion; RT, radiotherapy; W–D, well-differentiated; WBC, white blood cell. ^a^ Chi-square test; ^b^ Kruskal Wallis test (Z-test: WBC: 2.817; Lymphocyte: 45.737; Monocyte: 30.899; Albumin:52.928; LMR:116.366); * Personal habits include cigarette smoking, alcohol consumption, and betel nut chewing.

**Table 2:** Univariable and multivariable analyses of clinicopathological variables in relation to overall survival.

Variables		Univariable Analysis		Multivariable Analysis	
		HR (95% CI)	*p*-Value	HR (95% CI)	*p*-Value
Sex	Men vs. Women	1.546 (0.713–3.351)	0.270		
Age (years)	≥65 vs. <65	0.788 (0.510–1.218)	0.284		
cancer stage	III–IV vs. I–II	3.310 (1.904–5.756)	<0.001	2.066 (1.142–3.738)	0.016
Presence of PNI	Yes vs. no	2.344 (1.538–3.572)	<0.001		
Presence of LVI	Yes vs. no	3.827 (2.112–6.936)	<0.001	2.281 (1.191–4.369)	0.013
Tumor grading	P–D vs. W–D/M–D	2.934 (1.782–4.831)	<0.001	2.417 (1.424–4.043)	0.001
Closest margin (mm)	<5 vs. ≥5	1.445 (0.940–2.220)	0.093		
CCI	≥1 vs. 0	1.406 (0.934–2.116)	0.102		
mSIS	1 vs. 0	2.310 (1.462–3.617)	<0.001	2.021 (1.275–3.204)	0.003
mSIS	2 vs. 0	6.398 (3.627–11.284)	<0.001	5.095 (2.804–9.258)	<0.001

Abbreviations: CCI, Charlson comorbidity index; CI, confidence interval; HR, Hazard ratio; LVI, lymphovascular invasion; M–D, moderately differentiated; mSIS, modified systemic inflammation score; P–D, poorly differentiated; PNI, perineural invasion; W–D, well differentiated.

**Table 3:** Univariable and multivariable analyses of clinicopathological variables in relation to disease-free survival.

Variables		Univariable Analysis		Multivariable Analysis	
		HR (95% CI)	*p*-Value	HR (95% CI)	*p*-Value
Sex	Men vs. Women	1.442 (0.793–2.621)	0.232		
Age (years)	≥65 vs. <65	0.716 (0.505–1.017)	0.162		
AJCC stage	III–IV vs. I–II	2.066 (1.412–3.025)	<0.001	1.712 (1.137–2.578)	0.011
Presence of PNI	Yes vs. no	1.385 (0.962–1.996)	0.081		
Presence of LVI	Yes vs. no	1.994 (1.124–3.538)	0.018		
Tumor grading	P–D vs. W–D/M–D	2.094 (1.348–3.254)	0.001	2.024 (1.291–3.174)	0.002
Closest margin (mm)	<5 vs. ≥5	1.311 (0.925–1.857)	0.128		
CCI	≥1 vs. 0	1.064 (0.691–1.640)	0.776		
mSIS	1 vs. 0	1.489 (1.039–2.135)	0.030		
mSIS	2 vs. 0	3.904 (2.417–6.307)	<0.001	3.590 (2.167–5.947)	<0.001

Abbreviations: CCI, Charlson comorbidity index; CI, confidence interval; HR, Hazard ratio; LVI, lymphovascular invasion; M–D, moderately differentiated; mSIS, modified systemic inflammation score; P–D, poorly differentiated; PNI, perineural invasion; W–D, well differentiated.

## Data Availability

The data presented in this study are available upon request from the corresponding author and are subject to personal data protection regulations.
